# Operation and challenges of home-based medical practices in the US: findings from six aggregated case studies

**DOI:** 10.1186/s12913-018-2855-x

**Published:** 2018-01-27

**Authors:** Gregory J. Norman, Kristann Orton, Amy Wade, Andrea M. Morris, Jill C. Slaboda

**Affiliations:** West Health Institute, 10350 North Torrey Pines Rd, La Jolla, CA 92037 USA

**Keywords:** Home-based primary care, House calls, Home care, Team-based care, Geriatrics, Elderly, Frailty, Homebound, Qualitative study

## Abstract

**Background:**

Home-based primary care (HBPC) is a multidisciplinary, ongoing care strategy that can provide cost-effective, in-home treatment to meet the needs of the approximately four million homebound, medically complex seniors in the U.S. Because there is no single model of HBPC that can be adopted across all types of health organizations and U.S. geographic regions, we conducted a six-site HBPC practice assessment to better understand different operation structures, common challenges, and approaches to delivering HBPC.

**Methods:**

Six practices varying in size, care team composition and location agreed to participate. At each site we conducted unstructured interviews with key informants and directly observed practices and procedures in the field and back office.

**Results:**

The aggregated case studies revealed important issues focused on team composition, patient characteristics, use of technology and urgent care delivery. Common challenges across the practices included provider retention and unmet community demand for home-based care services. Most practices, regardless of size, faced challenges around using electronic medical records (EMRs) and scheduling systems not designed for use in a mobile practice. Although many practices offered urgent care, practices varied in the methods used to provide care including the use of community paramedics and telehealth technology.

**Conclusions:**

Learnings compiled from these observations can inform other HBPC practices as to potential best practices that can be implemented in an effort to improve efficiency and scalability of HBPC so that seniors with multiple chronic conditions can receive comprehensive primary care services in their homes.

## Background

The United States’ population of adults ages 65 and over is projected to double between 2005 and 2030, escalating the demand for medical care and increasing the national healthcare burden [1]. Those over age 80 are the most likely to be frail with multiple functional impairments and chronic conditions, and this population segment accounts for the highest health care consumption. In financial terms, it is well established that spending on health care services is highly concentrated among the highest consumers with just 5% of the US population accounting for 50% of health care spending in 2009 [2].

Chronic care for the frail elderly requires ongoing, low-intensity support, much of it not strictly medical [3], which is in stark contrast to acute care delivered in U.S. hospital systems. There are an estimated four million homebound seniors in the U.S. who need chronic care management and ongoing supportive services and account for a significant proportion of Medicare expenditures [4–6]. In the U.S., Medicare beneficiaries are considered homebound if they need the help of another person or medical equipment, such as a walker or wheelchair, to leave their home, and if their doctor believes their health could get worse if they leave home. Without easy access to primary care, homebound seniors resort to the emergency department and hospitalizations when they experience exacerbations of their chronic conditions [4].

Home-based primary care (HBPC) is a multidisciplinary ongoing care model for providing in-home treatment primarily to medically-complex, functionally impaired homebound seniors. Recent studies have demonstrated that HBPC can be a cost-effective strategy for delivering care to frail patients while maintaining or improving quality of care and patient satisfaction [7]. For example, a multidisciplinary team-based HBPC approach has been shown to increase physicians’ ability to see patients by 40%, reduce costs per patient by 20%, and maintain quality as well as patient and provider satisfaction compared to usual care [8]. A major advantage of long-term care provided in the home is that it enables the physician to evaluate the patient’s home environment, and be responsive to changes in health status, patient goals, and family caregiving capacity [9]. Using a case-study design, Muramatsu et al. concluded that primary care delivered in the home enhances quality of care, increases patient and caregiver satisfaction, and can replace the need for emergency and hospital visits [10]. Unfortunately, of the 2 to 4 million people in the U.S. who are homebound only about one quarter receives medical care at home [4, 11].

Overall, primary care home visits have steadily declined throughout most of Europe and North America since the mid-twentieth century, and are no longer considered part of usual care [12, 13]. By 2001, fewer than 18% of physicians in the U.S. made home visits. The decline of house calls in the U.S. can be attributed to the shift to managed care, and the pressure for providers to increase their productivity by seeing patients in centralized clinic settings, enabling them to see more patients each day [12]. However, the prevalence of physicians who make home visits has increased in the last decade in the U.S. following improved reimbursement for house calls by physicians [11, 12]. In Canada and Europe, physicians still make a sizeable number of home visits [12]. For example, in Germany, the traditional culture of house calls remains strong with GPs making a median of 6.5 home visits per week [13]. Many German GPs are self-employed, and competition to retain patients accounts in part for the motivation for patient home visits. There is consensus from physicians in Europe and North America that medical home visits are important for frail older adult patients to prevent unnecessary emergency department and visits and hospitalizations [13, 14].

Many HBPC practices in the U.S. are provider-led by a physician or nurse practitioner. The practice may also include a registered nurse, and medical assistants who support providers by triaging patients, assisting with patient intakes, and handling medication refills [8, 15]. Larger practices may employ administrative coordinators who provide scheduling, billing, procurement of supplies, and other administrative tasks; social workers who focus on the patient’s home environment and link patients to community supports and services; medical coders and billers; and transition nurses who facilitate the patient transfer from the hospital to the HBPC practice [16].

Home-base primary care patients in the U.S. primarily receive medical coverage through Medicare, in which care delivery is reimbursed on a fee-for-service basis for physician face-to-face patient visits [17]. This payment structure does not work well for coverage of a multi-discipline care team approach where care coordination outside of the home visit is necessary to meet the needs of HBPC patients [18]. In a study of the Mount Sinai Visiting Doctors HBPC program, it was estimated that 20.5% of providers’ time was spent on care coordination activities outside of home visits, and 2.4 h each week were not reimbursed [18]. When factoring in additional time spent during weekend hours, late nights, or on call, providers were unable to obtain reimbursement for nearly 4 to 8 h of care coordination each week. These large demands on providers, combined with the emotional toll of caring for frail seniors, may contribute to provider burnout and workforce shortages.

Our aim was to gain insight into HBPC programs across the U.S. by conducting a six-site HBPC practice assessment designed to better understand different structures, common challenges and approaches to address the complexities and complications of delivering ongoing care in the home. No single model of HBPC can be adopted widely without variation [3]. With this in mind, we aimed to learn what was working (and not working) across all six practices, which varied widely in terms of their business models, number of practice sites, practice locations in the United States, size, use of technology, and other factors.

## Methods

Site visits were conducted between July and August 2015 with six HBPC practices located across the U.S. Sites were identified using convenience sampling and selected based on each practice’s status as a home-based medical care provider and willingness to participate in on-site interviews. Leadership at eight practices were contacted about participation. Seven practices agreed to participate, however one practice was not able to be scheduled during the study period. The final sample consisted of five primary care practices and one telehealth monitoring program embedded in a health care system.

Site visits were conducted by at least two members of the study team. For each site visit, study team members spent one to two days directly observing in-office and field operations and conducting unstructured interviews with key members of the practice team, including care providers and individuals in administrative roles, to develop a better understanding of the practice’s organizational structure and current challenges. An unstructured qualitative approach was chosen for exploratory purposes and to facilitate a more open-ended guided conversation rather than a formal interview. Members of the practice discussed the operational structure of each practice in relation to four key areas: 1) Team composition: What is the team structure, and what are the roles and responsibilities of the team members? 2) Patient characteristics: How are new patients identified, selected, and enrolled into the program? 3) Use of technology: How is technology being used in home-based care? 4) Challenges: What are the challenges of HBPC practices? Field notes were taken during site visits to capture observations and insights. Interviews were not recorded.

At the conclusion of each site visit, study team members (KO and GN) de-briefed to gain consensus on their observations and field notes. One team member (KO), who participated in all six site visits, developed a set of detailed notes to facilitate cross-site comparisons. Notes were reviewed and further discussed by additional members of the study team (AM, AW, JS). Four study team members (AM, AW, GN, JS) identified cross-site themes and practice variations. This information was used to develop the aggregated case studies.

## Results

### Description of HBPC practices

Practices varied in terms of location, organizational structure, care team composition, specialized medical services provided, and practice size (Table 1). For simplicity we characterized a practice as small if its average census was below or equal to 600 patients, and large if the patient census was greater than 600. Four practices were affiliated with hospitals while the remaining two practices were stand-alone with no affiliation with any specific hospital or healthcare system. Of the four healthcare system-affiliated practices, one practice focused solely on home remote monitoring and conducted patient visits via video monitoring. The two stand-alone HBPC practices were very different in structure and operation. One practice had a central operations approach with multiple locations and operated with providers as independent agents accessing a core set of services and adhering to standardized performance measures. The second stand-alone HBPC practice was smaller and operated in one location.

**Table 1:** Practice characteristics

Practice	Region of United States	Organizational structure	Team composition	Practice size^a^	Patient average age	Provide urgent care
1	Northeast	House calls program integrated within an academic hospital system	Medical doctors, geriatrician fellow, transition nurse, coordinator	Small	77	No, but provide phone consultation and triage
2	Southwest	In home telehealth patient monitoring program as part of a non-profit healthcare system	Medical doctors, health coaches, nurses	Small	70	No
3	Northeast	House calls program integrated within an academic hospital system	Medical doctors, nurse practitioners, nurses, social worker	Small	85	Yes
4	Northeast	House calls program integrated within an academic hospital system	Medical doctors, nurse practitioners, nurses, medical coordinators, social workers, coder/biller	Large	82	Yes
5	West	Independent non-profit HBPC practice	Medical doctors, nurse practitioners, transition nurse, coder/biller, social worker, practice coordinator	Large	79	Yes
6	Pacific Northwest, Midwest, Southwest, Southeast	For-profit central service model with call center and independent regional offices	Medical doctors, nurses, practice coordinator, social worker, medical assistant, coder/biller	Large	74	Yes

^a^Practice size categorized as Small = ≤ 600 patients or Large = > 600 patients

The average patient age ranged from 70 to 85 across the practices. Aside from the remote monitoring practice, providers visited roughly three to eight patient homes a day, with total provider panels ranging from 100 to 200 patients.

#### Team composition and roles

**Fig. 1:**
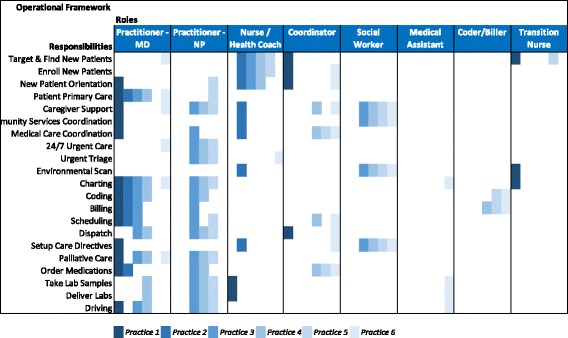
Care team configuration roles and responsibilities. Column headings represent the major team roles we observed. Within each role, the six practices are ordered within the column from left to right by increasing practice size. The rows list the tasks performed by care team members

All of the HBPC practices were composed of clinical care teams that were provider-led (physician or nurse practitioner), but the roles of these providers varied among the practices (Fig. 1). Smaller practices tended to be very provider-centered with a physician taking on multiple tasks, such as new patient orientations, scheduling, and ordering medications. Smaller practices did not have a social worker, medical assistant, or coder/biller. Large practices had a more diverse team and distributed tasks to specific team members, allowing the practitioner to focus more on primary care and palliative care.

#### How patients join a practice

Identifying the patients who can most benefit from HBPC and reduce expected costs of medically complex patients is a critical element of the HBPC model. We observed that patient inclusion differed from practice to practice based on resources, geographic constraints, practice size, referrals, revenue sources, organizational structure, and patient characteristics. However, there were some commonalities in identifying patients and referrals among the practices. Patients were identified either through self-selection, recognition of a new level of frailty, or through a referral from a primary care doctor aware of HBPC. It was common for patients to join HBPC practices after an acute event resulting in a hospital stay and a decline in health. Patients tended to be homebound with some practices using the Centers for Medicare and Medicaid Services’ definition of homebound as a requirement for eligibility. Finally, five of the six of practices required the patient to choose the HBPC practice as their primary care provider.

#### Technology and urgent care

In efforts to avoid hospitalizations in a medically complex patient population, four of the six practices provided urgent care visits and triage phone lines when emergencies occurred after normal business hours. If patients went to the hospital, five of the six practices received automatic hospital alerts. The remaining practice worked closely with local hospital staff to receive notifications when their patients were admitted to the hospital, and they were persistent in tracking the course of their patients’ care through the hospital system. Two practices used telehealth technology as a method to avoid hospitalization, which included community paramedics providing video teleconferencing with providers and real-time, continuous monitoring of vital signs. Another practice used mobile diagnostics (x-ray, ultrasound and phlebotomy) to track patient lab data over time and treat issues before they caused instabilities, avoiding hospital visits.

### Challenges

During observations and discussions with staff and providers at the practices, several challenges were stated that highlight the difficulty of delivering care to frail seniors in their home. These challenges were consistent across the practices and included provider retention, unmet demand for services, electronic medical records integration, and determining daily travel routes for providers.

#### Provider retention

When asked about challenges in operating a HBPC practice, individuals mentioned staff turnover and burnout. The emotional nature of treating a population of primarily frail, medically-complex patients near the end of life was one reason mentioned for staff turnover. Additionally, staff at the practices said the work can be lonely and isolating with days spent driving alone from home to home and time spent documenting care. Monetary compensation was a challenge for one practice since provider income was based on the number of patients visited, increasing provider case load in order to cover operational costs.

Although HBPC may be challenging, some providers mentioned aspects that kept them engaged in the profession. One provider stated that HBPC afforded a certain amount of autonomy and flexibility in their day as they were not constrained by standard clinic hours of operation. Another provider emphasized the benefit of spending time getting to know the patients, their families, and their home environment. Some providers stated they experienced a level of professional intimacy in treating patients in their home and enjoyed the impact they can have when the patient may have limited social connections outside of the home.

To help avoid provider burnout, a physician at one practice encourages providers to build a sense of connection with patients and learn more about their lives. Providers take photos of their patients during home visits and post them on boards in the office as a way to track physical changes in their patients over time, and to connect with patients on a personal level. The providers at the practice felt it was a healthy coping strategy that allowed providers to honor their patients’ lives and gain closure when patients die.

#### Unmet demand for services

One common concern voiced at all practices was that the need for HBPC in the community exceeded practice capacity. Most practices had long waitlists, and many expressed regret that sometimes they were compelled to turn away patients. The HBPC practices had different ways for dealing with demand. For example, one practice would only enroll patients within a limited number of zip codes based on driving distance. Another practice’s approach was to stratify patients based on hospital records and to offer services to patients with advanced illnesses first. Finally, several practices simply enrolled the next patient on the waiting list when a spot became available. In an effort to expand the workforce to meet demand, two HBPC practices partnered with medical schools to train geriatric and family medicine fellows in home-based care.

#### Electronic medical record (EMR) system integration

Providers can access their EMR from the field but these systems are not designed specifically for mobile providers, limiting the EMR’s functionality for home visits. EMR workflows are structured around a standard clinical setting where a patient is scheduled and seen at a specific time, neither of which is useful for home visits. Several practices we observed experienced challenges with their systems integration. Practices often purchased EMRs that were best suited for their needs and budget, however, these systems rarely integrated with EMR systems used by local hospitals or hospice centers.

#### Determining daily travel routes of providers

Practices varied in their methods for determining the daily travel route for providers. At some practices providers independently determined their routes, while at other practices travel routes were assigned to teams within a defined area. There were no technical solutions in place at any of the practices to automate and optimize routing of patient visits. While some practices used scheduling software, all of the practices handled logistics and routing manually. Computer optimized routing could increase the number of patients providers visit in a day and could potentially facilitate continuity of care for some patients. Even though commercial routing software exists and is routinely applied in manufacturing, shipping, and other industries, existing software packages do not integrate with EMRs and patient scheduling systems. Security measures would also be required for such a system to safeguard protected health information. Additionally, practices cautioned that it would be challenging to integrate a new software platform solely for their practice into a large medical system.

## Discussion

We visited six home-based medical practices that provide longitudinal care for medically complex seniors. Through observations and discussions with practitioners and staff we learned about each practice’s organizational structure, patient population, and challenges faced as a practice delivering medical care in the home. Combining observations from site-visits at six diverse practices delivering ongoing home-based medical care builds on previous single practice case studies [10, 12, 17, 18].

**Fig. 2:**
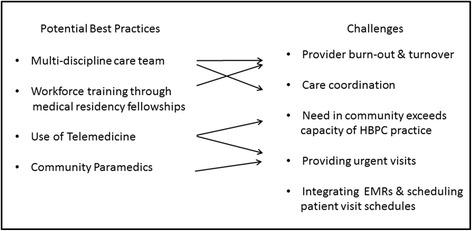
Challenges and Potential Best Practices for Delivering Home-based Primary Care

Learnings compiled from the site visits can inform other HBPC practices as to potential best practices that can be implemented in an effort to improve efficiency and scalability of HBPC without impinging on quality of care. Figure 2 lists four potential best practices that were in place at one or more of the practices. The arrows in the Fig. 2 point to the challenges each potential best practice can address. Further implementation research is needed to evaluate the use of telemedicine and community paramedics in home-based primary care. Figure 2 also highlights the need for technology solutions for integrating EMRs and optimizing patient visit schedules. Below we discuss the interconnections between the potential best practices and challenges of delivering HBPC.

Consistent with other reports, HBPC functions efficiently and has the best chance for scalability when it is practiced as team-based care [8, 15, 19]. We documented a variety of specialized roles important for delivering HBPC. These roles address the myriad of services HBPC practices provide their patients such as transitional care, care coordination, community-based services, palliative care, urgent care, and post-hospitalization follow-up. A recent review of the efficacy of care models for medically complex patients came to a similar conclusion that successful models of care include interdisciplinary teamwork, coordination of care, and facilitating transitions from hospital to post-acute care [20].

Unfortunately, it will be difficult for small practices to add personnel with specialized roles under reimbursement in fee-for-service Medicare. The smaller practices we visited were quite physician-centered in terms of staffing, with the physician taking on multiple roles and often not working at the top of their license. Taking on multiple roles and managing a large caseload of patients with a high mortality of 25–30% a year can lead to providers feeling isolated, frustrated and burned-out. In addition, full-time HBPC providers have been found to spend an average of 8.2 h per week providing care outside of home visits such as prescription refills, reviewing laboratory results, and coordinating care [18]. Most of this time does not get reimbursed in the current U.S. Medicare payment system. However, we noted that providers also find HBPC rewarding and have some autonomy in managing the hours they provide care. Providers who thrive in delivery of HBPC see it as an opportunity to really get to know their patients and deliver true patient-centered care.

We heard many patient stories from the practices depicting medical and non-medical needs of their patients. Patients were typically medically complex with multiple chronic conditions and social needs, homebound and often bed-bound. These are usually high cost patients who are not able to get to an outpatient clinic for ongoing care. HBPC is the appropriate care model for these patients from both the patient care and cost of care perspectives. DeJong and colleagues found the largest cost savings occurred when providing HBPC for patients identified with high levels of frailty [7].

Nearly all of the practices recognized that the need for home-based medical care in the community exceeds practice capacity as demonstrated by long waitlists for potential patients to join a practice. This is consistent with previous reports [8]. The need is exacerbated in rural areas where residents live considerable distances from the location of a HBPC provider [8]. There is a need for instruction in medical school and residency training on home-based medical care to meet the care needs of homebound patients [21]. We saw some practices creating opportunities for training fellows to experience medical home care, which may encourage new geriatric and family practice physicians to offer HBPC visits for their homebound patients. A HBPC rotation is an excellent opportunity for fellows to experience a house call practice and can contribute to developing the HBPC workforce [9, 17].

Methods of patient stratification based on disease acuity can help triage practice resources and patient workflows to provide higher touch and frequency of care to those patients in need, and lower frequency of care when patients’ conditions stabilize. As we observed, telemedicine for remote monitoring of vital signs and video consults can be an important tool for providing the needed level of care based on patient acuity. However, the evidence base for telemedicine integrated into home-based medical care has yet to be established [9].

Uses of technology such as telemedicine, portable diagnostic equipment, and lab testing are for the purpose of monitoring patients at home in an effort to detect anomalies before they become exasperations that require hospitalization or inpatient care. We heard how technologies such as automated hospital alerts and scheduling and routing systems could potentially increase the efficacy of running a HBPC practice. Unfortunately, these systems had limited to no diffusion in the practices we visited. Lack of implementation was often due to the fact that a practice was not directly integrated into a larger medical system. This was the case for practices without automated hospital alerts. On the other hand, practices embedded in a medical system were limited to EMRs and other system-wide software platforms that were not specifically designed for a mobile house calls practice. There is an opportunity to develop software information systems designed for HBPC practice operations, which can integrate with existing EMRs and have sufficient security and privacy standards to allow providers access outside the hospital.

Four of the six practices provided urgent care services for their patients, although practices require realistic projections of need to allocate scarce full-time employee resources. Using community paramedics to respond to urgent care calls is one innovative way to address the need, but there are challenges in how the visit is paid for when the patient is not transported to the hospital by the paramedics. Providers agreed that more often than not, when a patient or a caregiver calls with an urgent matter, they are able to resolve the problem over the phone. This highlights the value of patients and caregivers calling the practice rather than 911 to avoid an unnecessary emergency visit or hospitalization. A recent study showed providing 24/7 access to care was highly valued and considered a component of high quality HBPC by patients and caregivers [22]. Integrating community paramedics into the HBPC team provides a means of addressing urgent care needs and avoiding unnecessary hospitalizations [23].

Our findings should be interpreted with consideration of the study limitations. This was a series of observational case studies with a convenience sample of practices. The generalizability of the findings is limited to other similar types of medical practices. However, we anticipate that similar general themes would be identified at other HBPC practices. The findings may not generalize specifically to general practitioners who have patient encounters in clinic and patient home settings, which is more common in Europe [13]. There is the potential of information bias when conducting interviews where interviewees may want to provide favorable or self-promoting information about themselves or the practice. By spending at least a full day with each practice and interviewing multiple people at each practice, we learned both positive and challenging operational aspects about the practices. We did not collect financial, billing, or appointment information from the practices in order to minimize the burden to the practices participating in the site visits. In addition, medical practices can evolve over time for reasons ranging from staff turnover and internal process changes to mergers and restructuring. While the site visits capture a snapshot of six practices during a very specific window of time, our observations may no longer reflect the current structures or processes of the practices.

## Conclusions

The aggregated case studies revealed important operational issues concerning team composition, patient characteristics, use of technology, and urgent care for HBPC practices. Although there is no single model of HBPC, our findings address the need to identify and implement potential best-practice strategies, and bring to light barriers to delivering HBPC such as provider retention, meeting the demand for HBPC in a community, and integrating technology into practices. The lack of a sustainable Medicare payment model for adequately covering the cost of delivering multi-discipline team care has resulted in a serious gap in care for homebound medically complex seniors. The case studies highlight the potential for practice redesign methods and quality improvement strategies that can be implemented to stand-up, scale, and sustain a HBPC practice [4]. These efforts are more likely to succeed within a healthcare system with payment models that incentivize value over volume of care, and reimburse the team approach to HBPC, which encompasses much more than face-to-face home visits by a physician [18, 24].

## Abbreviations

EMR: Electronic medical record
HBPC: Home-based primary care
